# Peripheral blood inflammatory indexes in breast cancer: A review

**DOI:** 10.1097/MD.0000000000036315

**Published:** 2023-12-01

**Authors:** Jiaqiang Xie, Zhenxi Guo, Yijing Zhu, Mingde Ma, Guangwei Jia

**Affiliations:** a Department of Breast and Thyroid Surgery, Huaihe Hospital of Henan University, Kaifeng, Henan, China; b School of Clinical Medicine, Henan University, Kaifeng, Henan, China; c Department of Thyroid and Breast Surgery, Nanyang First People’s Hospital Affiliated to Henan University, Nanyang, Henan, China.

**Keywords:** breast cancer, lymphocyte-to-monocyte ratio, neutrophil-to-lymphocyte ratio, platelet-to-lymphocyte ratio, pan-immune-inflammation value, systemic immune-inflammation index, systemic inflammation response index

## Abstract

Immune and inflammatory responses play an important role in tumorigenesis and metastasis. Inflammation is an important component of the tumor microenvironment, and the changes in inflammatory cells may affect the occurrence and development of tumors. Complete blood count at the time of diagnosis and treatment can reflect the inflammatory status within the tumor. Studies have shown that the number of certain inflammatory cells in peripheral blood and their ratios are important prognostic factors for many malignancies, including neutrophil, lymphocyte, monocyte, and platelet counts, as well as neutrophil-to-lymphocyte ratio, platelet-to-lymphocyte ratio, lymphocyte-to-monocyte ratio, systemic immune-inflammation index, systemic inflammation response index and pan-immune-inflammation-value. The value of peripheral blood inflammation indexes in predicting the efficacy and prognosis of breast cancer neoadjuvant therapy is worth recognizing. This review details the application of peripheral blood inflammation indexes in the evaluation of efficacy and prediction of prognosis in neoadjuvant therapy for breast cancer, aiming to provide a more comprehensive reference for the comprehensive diagnosis and treatment of breast cancer.

## 1. Introduction

Breast cancer is the most common cancer among women worldwide and the leading cause of cancer-related death in women.^[1]^ Global cancer statistics for the year 2020 show that there were about 19.3 million new cancer cases worldwide. There are 2.3 million cases of breast cancer in these cases, accounting for 11.7%.^[1]^ The occurrence of breast cancer has been increasing year by year in recent years, and now its incidence and mortality rate have been ranked the highest among female cancers.^[1,2]^ As a result, breast cancer has become a major public health problem that poses a serious threat to human life and health and is receiving increasing attention. The exploration of therapeutic and predictive markers for breast cancer has also become a research hotspot.^[3–6]^ Traditional prognostic factors for breast cancer include lymph node status, tumor size, histological grade, pathological type, age, and race.^[7]^ In recent years, the role of inflammation and tumor microenvironment (TME) in cancer has been confirmed with the increasing understanding of the role and mechanisms of inflammation in the development and progression of malignancies.^[8]^ The TME is the environment around the tumor, which is mainly composed of tumor cells, immune cells, mesenchymal cells, extracellular matrix, signaling molecules, and cytokines.^[9]^ Tumors are closely related to the surrounding microenvironment and constantly interact with each other. Tumors can affect the TME by releasing extracellular signals, promoting tumor angiogenesis, and inducing peripheral immune tolerance, while immune cells in the TME can influence the growth and evolution of cancer cells.^[10,11]^ The immune cells in the TME mainly include neutrophils, monocytes, and lymphocytes. The measurement of peripheral blood inflammatory indexes can indirectly reflect the status of the tumor microenvironment. In the corresponding clinical studies, peripheral blood inflammatory indexes, including neutrophil-to-lymphocyte ratio (NLR), platelet-to-lymphocyte ratio (PLR), lymphocyte-to-monocyte ratio (LMR), systemic immune-inflammation index (SII), systemic inflammation response index (SIRI), and pan-immune-inflammation-value (PIV), have been used to evaluate the diagnosis and prognosis of malignant tumors.^[12–16]^ Therefore, the study of inflammatory indexes may provide clues for the diagnosis, treatment, and prognosis prediction of breast cancer.

## 2. Inflammation’s link to cancer

As early as the 19th century, German pathologist Rudolf Virchow already considered chronic inflammation as a factor in the origin of cancer.^[17]^ The link between cancer and inflammation has influenced cancer prevention and treatment for years.^[17]^ Later, studies have found that inflammation is involved in the stages of tumor initiation, progression, and invasion, and affects the prognosis of patients.^[17,18]^ Cancer cells interact with their surrounding stromal cells and inflammatory cells to form inflammatory TME which promotes tumor growth.^[19–24]^

### 2.1. Neutrophils

Neutrophils are one of the most abundant immune cells in the TME.^[25]^ Its interactions with other cells are critical to its function.^[26]^ Neutrophils have been reported to be associated with antitumor drug resistance.^[27–29]^ Recent studies have shown that neutrophil-driven antitumor drug resistance is dependent on interferon-gamma which is produced by T cells.^[30]^ Several studies have found that activated neutrophils can inhibit lymphocyte function by producing arginase-1 and hydrogen peroxide.^[31–33]^ Elevated neutrophil count will inhibit the secretion of tumor necrosis factor-alpha, leading to increased release of vascular endothelial growth factor (VEGF) in the blood circulation, and neutrophils are the main source of VEGF in the blood circulation. VEGF represents a growth factor with important proangiogenic activity, having a mitogenic and an anti-apoptotic effect on endothelial cells, increasing vascular permeability, promoting cell migration, etc.^[34]^ Due to these effects, it actively contributes to regulating the normal and pathological angiogenic processes. Therefore, overexpression of VEGF can promote tumor angiogenesis, which accelerates tumor growth and metastasis.^[35]^ In addition, neutrophils can release matrix metalloproteinase-9, neutrophil elastase, interleukin-8, and other inflammatory factors to promote tumor proliferation and metastasis.^[36,37]^

### 2.2. Monocytes

Monocytes are also the main immune cells of the body, and inflammation can trigger the activity of monocytes from the bone marrow to the peripheral blood.^[38]^ Peripheral blood monocytes can differentiate into tumor-associated macrophages (TAMs) after being recruited to tumor tissues by chemotaxis.^[39]^ Therefore, the number of circulating monocytes in the blood can indirectly reflect the number of TAMs. TAMs participate in tumorigenesis by secreting signaling molecules and extracellular vesicles.^[40]^ The cytokines and survival factors secreted by TAMs enhance the resistance of cancer cells to chemotherapy and radiotherapy.^[41]^ In addition, extracellular matrix deposition of TAMs promotes cancer cell resistance to chemotherapy and radiotherapy by remodeling or directing interactions between cancer cells and macrophages.^[42]^ TAMs promote tumor angiogenesis by upregulating VEGF levels.^[43,44]^ Furthermore, TAMs can also increase the production of angiogenesis-related growth factors by inducing proinflammatory mediators (e.g. IL-6 and IL-1).^[45]^ Hypoxia has been proven to be a key regulator of tumor angiogenesis. Under hypoxia, transcription of hypoxia-inducible factor-1α upregulates VEGF expression and promotes the proangiogenic function of TAMs.^[46]^

### 2.3. Lymphocytes

The number and percentage of lymphocytes in the body can reflect the current immune status of the body. TME releases chemokines that recruit peripheral blood lymphocytes to the tumor site, where they eliminate cancer cells by targeting tumor antigens and membrane ligands.^[47]^ Thus, alterations in the number of lymphocytes in TME affect the body’s antitumor response. Tumor-infiltrating lymphocytes (TILs) with anticancer properties are associated with less advanced cancers^[48]^ and better patient prognosis.^[49]^ Lymphocytes can induce cytotoxic death and inhibit tumor cell proliferation and migration,^[50,51]^ but their antitumor effects are related to the composition of the lymphocyte subpopulation in the TME. Cytotoxic CD8 (+) T cells are associated with a good prognosis. It is supported by CD4 (+) helper T cells that produce IL-2 and interferon-gamma, resulting in an effector mechanism that ultimately leads to tumor elimination.^[8,52,53]^ Cytotoxic lymphocytes recognize tumor-associated antigens and eliminate tumor cells by granule exocytosis (perforin and granzymes) and death ligands.^[54]^ However, Th17, a subpopulation of CD4 + T cells, can produce IL-17 in TEM. IL-17 is commonly associated with tissue inflammation and pro-tumor responses.^[55]^

### 2.4. Platelets

Platelets are an important part of the hemostatic process, but they have also been found to play a role in tumor progression and metastasis.^[56]^ Membrane receptors on platelet cell membranes can promote heterotypic cell interactions.^[57–59]^ These interactions play a key role in tumor growth and metastasis.^[58–60]^ It can also help tumor cell metastasis by releasing metalloproteinases. In addition, platelets can promote tumor and blood vascular growth by releasing inflammatory factors such as angiogenic factors, platelet-derived growth factors, and VEGF.^[60–62]^ Cancer cells that enter the circulation during metastasis will be exposed to the immune system. Cancer cells can use activated platelets to protect themselves from normal immune responses or natural killer cells,^[63,64]^ which contribute to tumor metastasis. In addition, cancer cells can directly or indirectly activate platelets and stimulate their aggregation,^[65,66]^ which is the reason why cancer patients have a higher risk of thrombosis.

## 3. Peripheral blood inflammatory indexes in breast cancer

### 3.1. NLR

NLR is calculated using NLR = N/L, where N and L are the pretreated peripheral neutrophil and lymphocyte counts, respectively. The level of NLR can reflect the situation of systemic inflammation and TME.^[67,68]^ Elevated levels of NLR significantly increase the risk of recurrence or death. In a long-term monitoring study of NLR changes in patients with triple-negative breast cancer (TNBC), it was found that elevated NLR during treatment suggested a worse prognosis. The results showed that the average preoperative NLR in the disease progression group was lower than that in the no evidence of disease group, but the NLR increased in the disease progression group during standard treatment, whereas it was stable or decreased in the no evidence of disease group.^[69]^ This result is similar to a previous study.^[70]^ In a recent meta-analysis, it was also confirmed that higher NLR was associated with poorer disease-free survival (DFS), overall survival (OS), and breast cancer-specific survival.^[71]^

Some studies have shown that changes in NLR during neoadjuvant chemotherapy (NAC) are associated with the efficacy of NAC and patient survival.^[68,72,73]^ This emphasizes the importance of monitoring the dynamic changes of indexes throughout the treatment process. Lou et al^[74]^ observed that high NLR is a risk factor for poor efficacy of NAC in TNBC patients and that high NLR may indicate poor prognosis in TNBC patients with failed NAC. Subsequent studies have confirmed this conclusion.^[75,76]^ In most adjuvant therapy studies, NLR was found to be an independent prognostic factor for survival. However, a meta-analysis of 45 studies showed that no significant correlation was found between survival and NLR for early breast cancer patients receiving NAC and advanced breast cancer patients.^[77]^ Li et al^[78]^ showed that a lower NLR (< 1.8) was significantly associated with a higher pathological complete response (pCR) rate and longer OS and that patients with a high NLR tended to have an increased lymph node metastasis rate. In addition, NLR has been found to predict axillary nodal pathologic complete response after neoadjuvant therapy for breast cancer.^[79,80]^

Philip et al^[81]^ found a strong correlation between NLR and pathological lymph node status in patients with TNBC (75% cases node-positive in the high NLR group vs 36% in the low NLR group). Additionally, NLR was found to be associated with clinical stage, but there was no significant correlation between it and OS. The study by Yang et al^[82]^ included 154 patients with cT1N0 breast cancer, 32 of whom had sentinel lymph node (SLN) metastases. According to univariate analysis, the SLN rate in the high level NLR group was higher than that in the lower group, but there was no statistical significance in multivariate analysis. These studies suggest that preoperative NLR has certain predictive value for the identification of benign and malignant breast tumors and axillary lymph node status, but it still needs to be further confirmed by studies with larger sample sizes.

### 3.2. PLR

PLR is defined as follows: PLR = P/L, where P refers to the pretreatment peripheral platelet counts. Patients with advanced breast cancer are often accompanied by elevated platelet counts, and higher PLR suggests worse OS and DFS.^[71]^ Moreover, PLR also showed prognostic relevance in ER + HER2-early breast cancer.^[83]^ An association has been found between high PLR rates and TIL immunosuppressive status in triple-negative breast cancer. Patients with high TIL/low PLR had a better prognosis than patients with low TIL/high PLR, and multiplex fluorescent immunohistochemistry showed that tumors in patients with high PLR and NLR contained more CD3CD4FOXP3 T cells.^[84]^ The study by Corbeau et al^[85]^ included 280 patients with early-stage breast cancer who underwent NAC. Multifactorial analysis showed that high PLR was an independent prognostic factor for shorter relapse-free survival and OS, but no significant association was found between PLR and pCR (*P* = .617). In contrast, another study that analyzed the PLR of 67 breast cancer patients receiving NAC treatment showed that patients with a high PLR (> 106.3) were significantly associated with better pCR than patients with a low PLR (< 106.3).^[86]^ However, a recent study found that the PLR before NAC in pCR group was lower than that in Non-pCR group (t = 3.290, *P* = .001).^[87]^ The main reason for this phenomenon may be because both studies were single-center, small-sample studies. This needs to be confirmed by a multicenter, large sample-size study. Before collecting peripheral blood, interference of infection, bleeding, immune diseases, and other factors were excluded as much as possible.

In a study involving 202 patients with early-stage breast cancer, the risk of a positive SLN was found to be 0.43-fold higher in patients in the high PLR group (> 139.45) than in the low PLR group (< 139.45).^[88]^ A statistically significant correlation was also found between PLR and the number of metastatic lymph nodes, with a 1-unit increase in PLR leading to a 0.134-unit increase in the number of metastatic lymph nodes (rho = 0.199, *P* = .004).^[88]^ In patients with cT1N0 breast cancer, the risk of SLN metastasis was also found to be higher in patients with high levels of PLR than in patients with low levels of PLR.^[82]^ However, it has also been reported that the PLR of patients with non-SLN metastasis is lower than that of patients without non-SLN metastasis.^[89]^ The above studies illustrate that PLR has some value in the prognosis of breast cancer patients, but remains controversial in predicting lymph node status and NAC efficacy. Combined with other inflammatory markers may be able to more accurately predict the prognosis of breast cancer patients.

### 3.3. LMR

LMR is calculated using L/M, where M is the pretreatment peripheral monocyte counts. In a study that included 114 patients with HER2 negative advanced breast cancer, it analyzed the predictive value of pretreatment LMR for paclitaxel in combination with bevacizumab. The results showed that patients with high LMR had a longer time to treatment failure and OS.^[90]^ In a single-center retrospective study that included 440 breast cancer patients with disease progression in 224 (51%) and death in 62 (14%) after a median follow-up of 72.9 months, Kaplan–Meier survival curve analysis showed that higher LMR (≥ 4.85) was associated with longer median DFS (median DFS, 85.83 vs 60.90, *P* < .001).^[91]^ In addition, high LMR is also associated with better prognosis in ER + HER2-early breast cancer.^[83]^ Ma et al^[92]^ investigated the relationship between LMR and breast cancer before NAC treatment, including 203 breast cancer patients. The results showed that low LMR was significantly associated with lymph node metastasis and clinical T-stage, and NAC patients with low LMR showed higher pCR rates and better chemotherapy effects. Kaplan–Meier survival curves indicated that patients with low LMR had poorer DFS. In another study, the prediction of pCR by LMR showed the opposite results in breast cancer patients receiving NAC. The study included 241 patients, of whom 48 (19.92%) achieved pCR after NAC treatment. The results showed that 33 (16.41 %) of 201 people in the low LMR group achieved pCR (< 5.38), and 15 (37.50 %) of 40 people in the high LMR group achieved pCR (≥ 5.38), but there was no statistical significance in multivariate analysis (*P* = .437).^[93]^ The above studies suggest that LMR has an important predictive value for the prognosis of breast cancer patients. However, there is no uniform delineation of the cutoff value and some controversy remains in the prediction of pCR in breast cancer patients receiving NAC. This needs to be further validated in a larger multicenter prospective clinical study. Also of interest is monocyte-to-lymphocyte ratio, which has prognostic potential for breast cancer patients who receive NAC.^[94]^

### 3.4. SII

SII which is calculated based on 3 blood cell counts may be a more balanced index of the inflammatory state of the organism compared to NLR and PLR. It is defined as follows: SII = (P × N)/L. SII reflects the balance between host inflammation and immune status. At present, more and more studies have shown that SII is a clear prognostic factor for a variety of malignancies. Its application in breast cancer has also been increasing in recent years. Some studies have shown that SII is superior to NLR and PLR in predicting the prognosis of breast cancer patients.^[95,96]^ In a study involving 262 breast cancer patients receiving NAC, patients in the low SII group (< 602) had longer DFS and OS than those in the high SII group (> 602) (40.76 vs 31.11 months; 53.68 and 44.47 months, respectively).^[97]^ High SII also tends to predict poor survival in patients receiving adjuvant trastuzumab therapy.^[98]^ Preoperative SII has also been found to be a prognostic predictor in breast cancer patients undergoing surgery. A study that included 784 breast cancer patients who underwent surgical resection found high SII to be a poor prognostic factor for DFS and OS according to Kaplan–Meier survival curve analysis.^[99]^ The study also analyzed the relationship between SII and clinicopathological characteristics and found that SII was also significantly associated with younger age, positive PR expression, and positive HER2 expression. In a retrospective case-control study of 560 patients with early invasive breast cancer, increased SII was found to predict an increased risk of non-SLN metastasis, and elevated SII served as an independent predictor for non-SLN metastasis following positive SLN.^[100]^ A recent study came to a similar conclusion.^[101]^ Moreover, high SII levels are associated with endocrine therapy resistance in patients with luminal breast cancer.^[102]^

### 3.5. SIRI

SIRI is an effective index of the immune status of malignant tumors that is established on peripheral venous lymphocyte, monocyte, and neutrophil counts.^[103]^ SIRI is calculated using (N × M)/L.^[15]^ Wang et al^[104]^ explored the prognostic value of SIRI before and after surgery in operable breast cancer patients and found that breast cancer patients with low SIRI (≤ 0.65) had significantly higher OS than those with high SIRI (> 0.65). In addition, this study showed that the change in SIRI at 4 weeks after surgery was strongly associated with survival in breast cancer patients, and breast cancer patients with larger increases in SIRI scores had worse OS.^[104]^ In a retrospective study that included 390 postmenopausal breast cancer patients undergoing mastectomy, high SIRI (> 0.54) was found to be significantly associated with poorer prognosis and progesterone receptor status, and SIRI was an independent predictor of OS according to a multifactorial analysis of this study.^[105]^ In breast cancer patients undergoing NAC, it has been shown that pretreatment SIRI is superior to LMR as a prognostic indicator.^[106]^ Another retrospective study involving 262 breast cancer patients who received neoadjuvant chemotherapy found that patients with low SIRI had longer DFS and OS than those with high SIRI (41.27 vs 30.45 months; 52.86 vs 45.75 months, respectively) and better DFS and OS at 3, 5, and 10 years than those with high SIRI.^[107]^ In a retrospective analysis, pretreatment SIRI was found to be significantly associated with pCR in breast cancer patients receiving NAC. The low SIRI group (< 0.72) was nearly 5-fold more likely to achieve pCR than the high SIRI group (> 0.72), and SIRI was shown to be an independent prognostic factor for pCR in breast cancer patients in a multifactorial analysis.^[93]^

### 3.6. PIV

PIV is a recently developed comprehensive index which is defined as follows: PIV = (N × M × P)/L. PIV was first applied to evaluate the prognosis of advanced colorectal cancer, and the results showed that PIV was a strong predictor of metastatic colorectal cancer.^[16]^ In a recent meta-analysis that included multiple cancers, including 15 studies with a total of 4942 patients, the results showed that patients with higher PIV had a markedly increased risk of disease progression and death compared to those with lower PIV.^[108]^ This finding was also confirmed in a recent study.^[109]^

pretreatment PIV seems as a predictor for pCR and survival, outperforming NLR, monocyte-to-lymphocyte ratio, and PLR in predicting pCR.^[110]^ A Turkish retrospective study that included 743 breast cancer patients receiving NAC showed that patients with low PIV had better responses to chemotherapy and that patients with low PIV were significantly associated with longer DFS and OS (*P* = .034, *P* = .028, respectively).^[110]^ And, the results of a recent multicenter study involving 1274 patients treated with NAC also confirmed that low PIV independently predicts an increased likelihood of lymph node pCR.^[80]^ In operable breast cancer patients, PIV showed the same results for predicting prognosis.^[111]^ Another retrospective study including 57 patients with HER2-positive breast cancer showed that high PIV was associated with poorer PFS and OS in patients treated with first-line taxane-trastuzumab-pertuzumab.^[112]^ PIV is a more comprehensive inflammatory index, which is superior to other inflammatory indexes in assessing the prognosis of patients. In contrast to other inflammatory indexes, PIV combines all routinely assessed blood cell populations that reflect systemic inflammation and immunity.^[109]^ As such, it provides a more complete picture of the host’s condition and is of more reliable value for prognostic assessment. However, there are few related studies on it, and more research is needed to explore it further.

## 4. Conclusion

In summary, the present study indicates that the application value of peripheral blood inflammatory indexes in breast cancer is worthy of affirmation. Complete blood count is one of the routine examinations for breast cancer patients, which has the advantages of simplicity, economy, and repeatability. It is more convenient and less expensive than imageological examination, pathological examination, and blood markers. Combining inflammatory indexes such as NLR, PLR, LMR, SII, SIRI, and PIV with other indicators may provide additional references for breast cancer assessment and treatment strategy formulation. However, the methods for obtaining the optimal cutoff value vary among studies, and there is a lack of consensus on the optimal cutoff value of each inflammatory index. And, the populations targeted by the published studies vary. Moreover, most of the current studies are single-center retrospective studies, and there are some controversies. Large prospective studies are still needed to determine the true clinical value and applicability of the inflammatory indexes before the peripheral blood inflammatory indexes can be widely used as clinical predictive indicators.

## Author contributions

**Conceptualization:** Mingde Ma, Guangwei Jia.

**Data curation:** Jiaqiang Xie, Zhenxi Guo, Yijing Zhu, Mingde Ma, Guangwei Jia.

**Formal analysis:** Jiaqiang Xie, Zhenxi Guo, Yijing Zhu, Mingde Ma, Guangwei Jia.

**Funding acquisition:** Mingde Ma.

**Investigation:** Jiaqiang Xie.

**Methodology:** Jiaqiang Xie.

**Project administration:** Mingde Ma.

**Supervision:** Mingde Ma, Guangwei Jia.

**Writing – original draft:** Jiaqiang Xie.

**Writing – review & editing:** Jiaqiang Xie, Mingde Ma, Guangwei Jia.
